# Micropropagation and Production of Health Promoting Lignans in *Linum usitatissimum*

**DOI:** 10.3390/plants9060728

**Published:** 2020-06-09

**Authors:** Irfan Khan, Mubarak Ali Khan, Muhammad Amir Shehzad, Amir Ali, Sher Mohammad, Huma Ali, Mohammed Nasser Alyemeni, Parvaiz Ahmad

**Affiliations:** 1 Department of Biotechnology, Faculty of Chemical and Life Sciences, Abdul Wali Khan University Mardan (AWKUM), Mardan 23390, Pakistan; ibnefazalkhanbb2@gmail.com (I.K.); shehzadaamir946@gmail.com (M.A.S.); 2 Biotechnology Lab. Agricultural research institute (ARI), Tarnab, Peshawar 25000, Pakistan; amirkhan31530@gmail.com (A.A.); drshermohammad@gmail.com (S.M.); 3 Department of Biotechnology, Bacha Khan University Charsadda, Peshawar 24420, Pakistan; write2humaali@gmail.com; 4 Botany and Microbiology Department, College of Science, King Saud University, Riyadh 11362, Saudi Arabia; mnyemeni@ksu.edu.sa

**Keywords:** lignans, *Linum usitatissimum*, thidiazuron, antioxidants, in vitro cultures

## Abstract

*Linum usitatissimum* commonly known as flax or linseed is an important medicinal plant, produces medicinally potent lignans, used in the treatment of several human diseases. Lignans limited production in the natural plants does not meet the increasing market demand. This study was conducted to establish an easy and rapid method for the in vitro micropropagation and production of potent lignans and antioxidant secondary metabolites in linseed. The results indicated that hypocotyl explants under the effects of thidiazuron (TDZ: 0.5 mg/L) + kinetin (Kn: 0.5 mg/L) in the basal growth media, resulted in the optimal shoot organogenesis parameters (shoot induction frequency: 86.87%, number of shoots: 6.3 ± 0.36 and shoots length: 6.5 ± 0.54 cm), in 4 weeks. Further, TDZ supplementation in the culture media efficiently activated the antioxidant system in the in vitro raised shoots, wherein maximum production of total phenolic content, TPC (34.33 ± 0.20 mg of GAE/g DW); total flavonoid content, TFC (8.99 ± 0.02 mg of QE/g DW); DPPH free radical scavenging activity (92.7 ± 1.32%); phenylalanine ammonia-lyase activity, PAL (8.99 ± 0.02 U/g FW); and superoxide dismutase expression, SOD (3.62 ± 0.01 nM/min/mg FW) were observed in the shoot cultures raised in presence of TDZ: 0.5 mg/L + Kn: 0.5 mg/L. Nonetheless, considerable levels of pharmacologically active lignans such as secoisolariciresinol (SECO: 23.13–37.10 mg/g DW), secoisolariciresinol diglucoside (SDG: 3.32–3.86 mg/g DW) and anhydrosecoisolariciresinol diglucoside (ANHSECO: 5.15–7.94 mg/g DW) were accumulated in the regenerated shoots. This protocol can be scaled up for the commercial production of linseed to meet the market demands for lignans.

## 1. Introduction

*Linum usitatissimum* commonly known as linseed or flax is an important medicinal plant belongs to the genus *Linum* and family Lianaceae [1,2]. Linseed has been cultivated since 5000 BC in Mediterranean belts, including Europe and Asia. In Pakistan, linseed locally named as Alsi, is found in Punjab, Khyber Pakhtunkhwa and Sindh provinces [3]. Morphologically, it is an herbaceous plant, which has a smooth, erect stem and shallow root system, and has various secondary branches. The seed capsule is known as seed bolls. Seeds may be golden (Bionda) or brown (Antares) in color [4]. Major components of the linseed envelope or testa are fats, proteins, mucilage, and dietary fiber. Approximate composition of linseed plant represents 41% fat, 20% protein, 28% TDF (total dietary fiber), 7.7% moisture and 3.4% ash [5,6].

Linseed oil is a rich source of functional bioactive metabolites such as omega-3-fatty acid [7], omega-6-fatty acid, and linoleic acid, which are polyunsaturated fatty acids and are useful for prevention and managing critical and chronic disorders like Alzheimer’s disease, rheumatoid arthritis, high blood pressure, type 2 diabetes, coronary heart disease, and stroke, etc. [8]. Linseed also contains some medicinally potent, phenolic acids and flavonoids [9]. However, the major bioactive compounds are lignans also known as phytoestrogens as they regulate and maintain the estrogen hormonal level in the human body [10]. Lignan is a phyto-polymer present in the conductive xylem tissue of linseed that gives stiffness and strength to the stem and reduces cell wall permeability to prevent pathogens attack [11].

Linseed is a rich source of lignans and neolignans and contains 800 times more lignans than other medicinal plants [11]. SDG (secoisolariciresinol diglucoside), MAT (matairesinol), SECO (secoisolariciresinol), and LDG (lariciresinol diglucoside) are the major lignans produced in linseed, reported for their beneficial health effects in the prevention and cure of different cancers like breast, colon, and prostate cancer [11,12]. *L. usitatissimum* also contains both water and fat-soluble vitamins such as vitamin E (tocopherols). Vitamin E has antioxidant potential of preventing the cell proteins and fats from oxidation, increasing the excretion of sodium in the urine, decreasing the risks of cardiovascular disease by lowering the blood pressure, and also acting as an anti-cancer agent in cure of Alzheimer’s disease [13,14].

*L. usitatissimum* is a rabi seasonal plant and can efficiently grow in the moderate temperature regions. Its growth and yield are adversely affected by scarcity (drought) and hot environmental conditions [3]. Soil texture and pests manifestation are the other factors that can limit the production of linseed during routine cultivation procedures [15]. Similarly, linseed is prone to all infectious agents such as bacteria, fungi, and viruses. *Alternaria* blight, powdery mildew rust, and wilt are the notable diseases infecting linseed [10]. The demand of linseed in food industries has rapidly increased recently because of its new prospective as a functional food due to its significant nutritional and medicinal applications [15]. The increasing market demand can be met by the rapid multiplication technologies (RMTs) such as in vitro germination, micropropagation, and cell and callus cultures under controlled laboratory conditions [16]. Establishment of plant in vitro cultures through optimization of culture conditions and hormonal media might directly influence plant cell viability, growth, regeneration, and biomass formation as well as the aseptic maintenance of plant germplasm for longer periods [17]. Cytokinins and auxins are the principal plant growth regulators (PGRs), which are highly recommended for the feasible micropropagation in diverse medicinal plants [18]. 1-phenyl-3-(1,2,3-thiadiazol-5-yl)-urea, or TDZ, is amongst the most influential PGRs in the regeneration of many industrially important plants. The morphogenetic regulation by TDZ in several medicinal plants has influenced the feasible growth and development due to its dual function of that a cytokinin and an auxin hormones [19].

TDZ is reported for its direct impact on the antioxidant system of plant cell through activation of the anti-stress elements such as the enzymatic and non-enzyme components to mitigate the stress conditions, and consequently they help the plant cell to continue normal growth and development [20,21]. The main objectives of this study were to analyze the impacts of TDZ on the in vitro micropropagation, secondary metabolism, and biosynthesis of bioactive lignans in *L. usitatissimum* through analysis by gas chromatography-mass spectrometry (GC-MS).

## 2. Results and Discussion

### 2.1. Optimization of the Suitable Explant and Plant Growth Regulators (PGRs) for In Vitro Shoot Induction

The main objectives of this study were to develop a rapid method for micropropagation and production of bioactive secondary metabolites in *Linum usitatissimum*. In the preliminary experiments, seeds of linseed were in vitro germinated on MS0 media for a continual supply of aseptic explants, used in subsequent experiments for micropropagation. Optimum seed germination frequency (84%) was recorded in the culture tubes after seven days of culture. For continual supply of different explants, in vitro seed germination has been proved as an effective strategy to provide an aseptic germplasm as a source of explants and may inevitably reduce the chances of contamination. Usually the explants derived from the in vitro raised plantlets are more resistant to the microbial contamination during plant tissue culture studies, as compared with explants taken from the field grown plants [22]. In order to determine and select the suitable type of explant for micropropagation in this study, four different explants including leaf, cotyledon, root, and hypocotyl were cultured on MS media augmented with different concentrations of TDZ and Kn alone or in combinations. The shoots were induced in almost all explants but with variable frequencies. Highest shoot induction frequency (86.87%) was observed in the hypocotyl explants incubated on MS media containing TDZ (0.5 mg/L) + Kn (0.5 mg/L). TDZ alone also resulted in maximum shoot induction (86.54%) when added at 0.5 mg/L in the culture media. Leaf and cotyledon explants resulted in 59.43% and 56% shoot induction frequencies respectively from the culture media supplemented with TDZ (1.0 mg/L) + Kn (1.0 mg/L). However, lowest response (45.76%) was observed for root explants (Table 1).

During plant cell in vitro growth, the nature of the explants plays a prominent role and determines the type and mode of regeneration, for instance, callus induction, shoot formation, induction of adventitious roots, or somatic embryos. However, the regeneration potential in any plant species is strictly dependent on the availability of growth regulators, media composition, plant genotype, and the physiological status of the explant [23,24]. In another article, hypocotyl explants are reported for their pertinent role during shoot organogenesis of linseed [25].

In this study, PGRs such as TDZ and Kn when employed solitary in the MS media at different levels were found less effective for shoot induction as compared with treatments of their combinations (Table 1). Likewise, hypocotyl explants of linseed (Mikael variety) produced optimum adventitious shoots (82% and 100%) in MS0 (control) or PGRs (2.0 mg/L TDZ + 0.1 mg/L NAA) supplemented media, respectively [26]. Similarly, hypocotyl explants of *L. usitatissimum* induced about 9.1 buds per explant in MS media fortified with 1.0 ppm Kn [27].

Janowicz et al. [25] reported that 92% shoots were induced from the hypocotyl explants of linseed in MS + 1.0 BAP media. Furthermore, Akter et al. [28] reported that 69% linseed shoots were induced from internode explant cultured in media supplemented with 2 mg/L BA + 0.5 mg/L NAA. Our data showed that lower concentrations of both TDZ and Kn attributed to the optimum response of shoot induction from the hypocotyl explants, wherein, high concentration of PGRs (TDZ and Kn) indicated lower induction response (Table 1).

Studies have shown that TDZ at higher concentrations produce isopentenyl adenine (iP) which promotes cell division. The formation of iP via high level of TDZ might results in callus initiation instead of shoot formation in explants [29,30]. Further, it was inferred that TDZ might increase the level of endogenous cytokinins through inhibition of cytokinin oxidase enzyme, which catalyzes the inactivation of the cytokinins [20,31].

### 2.2. TDZ Mediated Micropropagation and Growth Parameters

Significant variations in shoot induction, multiplication and growth parameters were observed in this study (Table 2). The shoots were directly induced in the hypocotyl explant in all treatments. Naggar et al. [27] observed direct buds formation on the surface of hypocotyl explants cultured in MS media supplemented with 1.0 ppm Kn without any callus formation in *L. usitatissimum*. In a similar study, Ali et al. [32] reported direct shoot regeneration in *Ajuga bracteosa* in response to TDZ (0.5 ppm).

**Figure 1:**
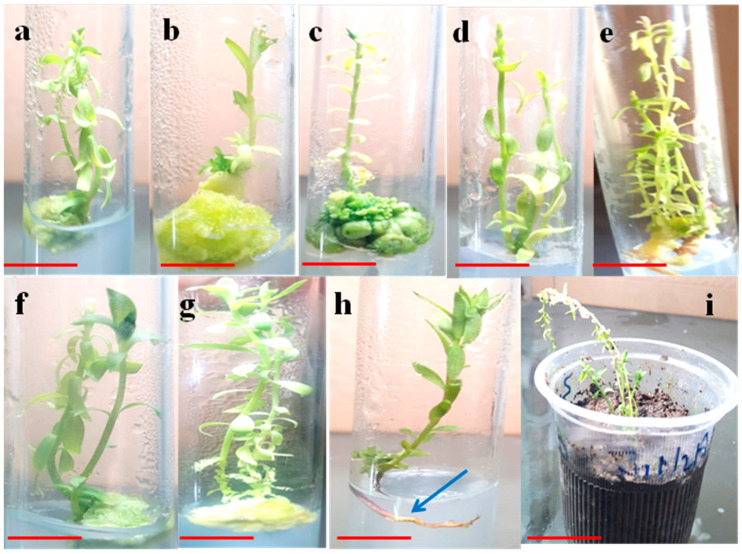
Invitro micropropagation in *L. usitatissimum,* (**a**): shoot regeneration in hypocotyl explants in response to 0.5 mg/L TDZ, (**b**): shoot regeneration in hypocotyl explants in response to 1.0 mg/L TDZ, (**c**): shoot regeneration in hypocotyl explants in response to 1.5 mg/L TDZ, (**d**): shoot regeneration in hypocotyl explants in response to 0.5 mg/L Kn, (**e**): shoot regeneration in hypocotyl explants in response to 0.5 mg/L TDZ + 0.5 mg/L Kn, (**f**): shoot regeneration in hypocotyl explants in response to 1.0 mg/L TDZ + 0.5 mg/L Kn, (**g**): shoot regeneration in hypocotyl explants in response to 1.5 mg/L TDZ + 0.5 mg/L Kn, (**h**): rooting of the micropropagated shoots in presence of 0.1 mg/L IBA at half strength MS media, arrow indicates the development of roots at the base of shoots, and (**i**): hardening and acclimatization of the micropropagated plants, Bar 2.0 cm.

In our study, early or first shoots were induced on day fifth after inoculation in hypocotyl explants in the solitary TDZ (1.0 mg/L) treated cultures but in the presence of Kn (0.5 mg/L) the shoots induction took more time (10 days) (Table 2). In a similar study, TDZ supplementation (7 μM) in the culture media favored rapid shoot regeneration in Mulberry plant [33]. In this study, maximum number of shoots (6.3 ± 0.36) with higher length (6.5 ± 0.54 cm) were observed in the basal media containing TDZ (0.5 mg/L) + Kn (0.5 mg/L) (Table 2; Figure 1e), wherein the lowest shoot number (1 ± 0.11) and shoot length (3.3 ± 0.28 cm) were recorded in the TDZ (1.0 mg/L) treated cultures (Figure 1b).

In several studies TDZ has shown better response in shoot regeneration than BA, 2 4-D and NAA [25,34,35]. For instance, TDZ (2.0 mg/L) in combination with NAA (0.1 mg/L) resulted in regeneration of multiple shoots (15.3 ± 0.88) in stem explants of *L. usitatissimum* [26]. Similarly, considerable number of shoots were regenerated in linseed explants grown in presence of 2,4-D (2 mg/L) and BAP (1 mg/L) [36].

In a similar study, Khan et al. [20] reported maximum shoots per explants in *Silybnum marianum*, cultured in media containing 11 μM TDZ. Our findings indicated that TDZ in combination with Kn showed optimum shoot regeneration as compared with individual TDZ treatments. However, lower doses of TDZ and Kn attributed better response in shoot regeneration than higher concentrations. Similarly, TDZ with other PGRs has been reported as more effective and inductive in shoot organogenesis than solitary TDZ application in some important medicinal plants [37]. When tested alone, TDZ induced 80% shoots regeneration in chicory. However the shoot regeneration potential was incremented further up to 95% when TDZ (1.0 mg/L) was combined with IAA (1.0 mg/L) in the culture media [38]. Similarly, TDZ alone in comparison to conventional PGRs such as IAA and BA, was comparatively more efficient growth regulator in shoot morphogenesis in *Kigelia pinnata*, *Cichorium intybus* and *Phaseolus vulgaris* species [33,38,39].

TDZ is considered to energize the endogenous cytokinins by activating the transition of cytokinin nucleotides as more effective and efficient biological nucleotides [40]. In this study, callus biomass formation was also observed in hypocotyl explants at higher concentration of TDZ wherein at (1.5 mg/L) TDZ alone produced maximum callus mass (1033 ± 11.01 mg FW) (Table 2; Figure 1b,c). Available reports have pointed out with clear notion that TDZ and cytokinins share a similar mode of action in plants [20,32]. Low levels of TDZ (0.0 to 0.005 μmol/L) induce the formation of zeatin riboside (ZR) while at higher concentrations (≥0.5 μmol/L) it produces isopentenyl adenine (iP), which regulates the cell division. The production of ZR at low levels of TDZ ultimately results in the shoot regeneration and multiplication. However, the formation of iP at higher TDZ doses favors callus formation in explants [29,30]. The shoot multiplication potential of TDZ as indicated in this study might be paralleled to its cytokinin like activity in which buds were directly induced laterally in the stem explants of linseed [41] as well as in *Ajuga bracteosa* [32].

At elevated levels TDZ is reported for callogenesis as it suppresses bud initiation and bud breaking thus ultimately reduces the shoot induction frequency [41]. Furthermore, in TDZ treated cultures, high levels of endogenous auxin, ethylene, and ABA were witnessed during callus formation [42,43,44]. Our results indicated that optimum callogenesis occurred at high TDZ concentration while at low level less callus formation was observed. Nonetheless, more callus formation was reported in *Linum usitatissimum* from cotyledon explant as compared to hypocotyls in MS media augmented with 1.0 mg/L Kn [26].

### 2.3. Roots Formation and Acclimatization

The in vitro propagated shoots were then inoculated in the MS media with or without PGRs for rooting. A modified MS media was used for rooting of the shoots because of very low rooting response in response to TDZ at different concentrations. As indicated in Table 3, the results showed that roots formation was initiated in shoots grown in half strength MS media augmented with 0.1 mg/L IBA. Wherein, the highest growth responses during in vitro rooting (induction frequency: 45.7 ± 2.54%, root length: 3.6 ± 0.46 cm) were observed, respectively. While the lowest response was observed at full strength MS media supplemented with 1.0 mg/L IBA. In different reports, IBA either alone, or in combination with other PGRs, have positively influenced root induction in many medicinal plants. Both IBA and NAA are the synthetic PGRs reported for higher root induction frequency in *Orthosiphon stamineus* [45,46].

During in vitro cultures of plants, IBA has shown an effective role on the production of ethylene, which stops bud opening for shoot regeneration and thus activates the roots formation in shoots [47]. Finally, the well-developed healthy plants were uniformly transferred to the vermiculture and placed in the growth chamber till successful acclimatization. Then after five weeks the plants were shifted to the field conditions in which above 75% of plants survived with normal growth rate and no phenotypic or morphogenetic abnormalities.

### 2.4. Production of Antioxidant Secondary Metabolites in Linseed

Different factors such as light, PGRs, and elicitors, not only affect shoot regeneration and biomass formation but also influence on the production of different vital secondary metabolites during micro propagation [48]. Plant cell synthesizes these secondary metabolites under stress conditions. Stress condition is created by the over production of different singlet oxygen moieties, also called reactive oxygen species inside plant cell which sometimes causes cell necrosis or cell death [49]. Different classes of secondary metabolites for instance flavonoids, phenolic compounds, glycosides, alkaloids, terpenoids, etc., are produced by the plant cell to mitigate the harsh stress conditions and continue normal growth and development [50].

**Figure 2:**
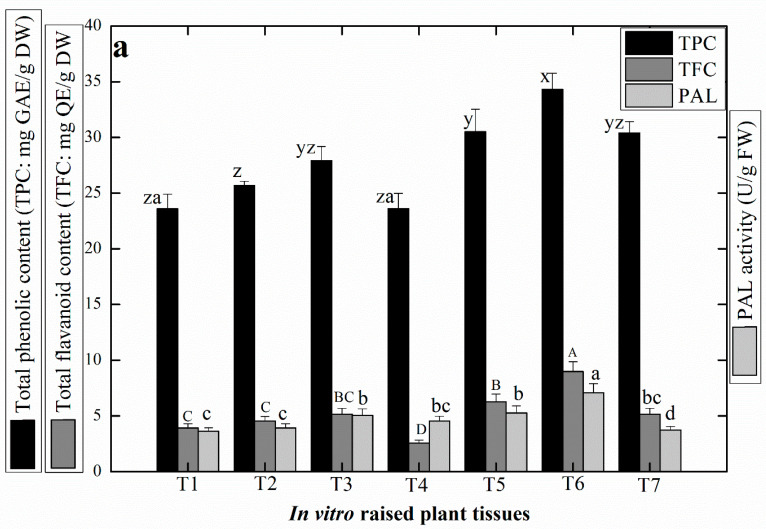

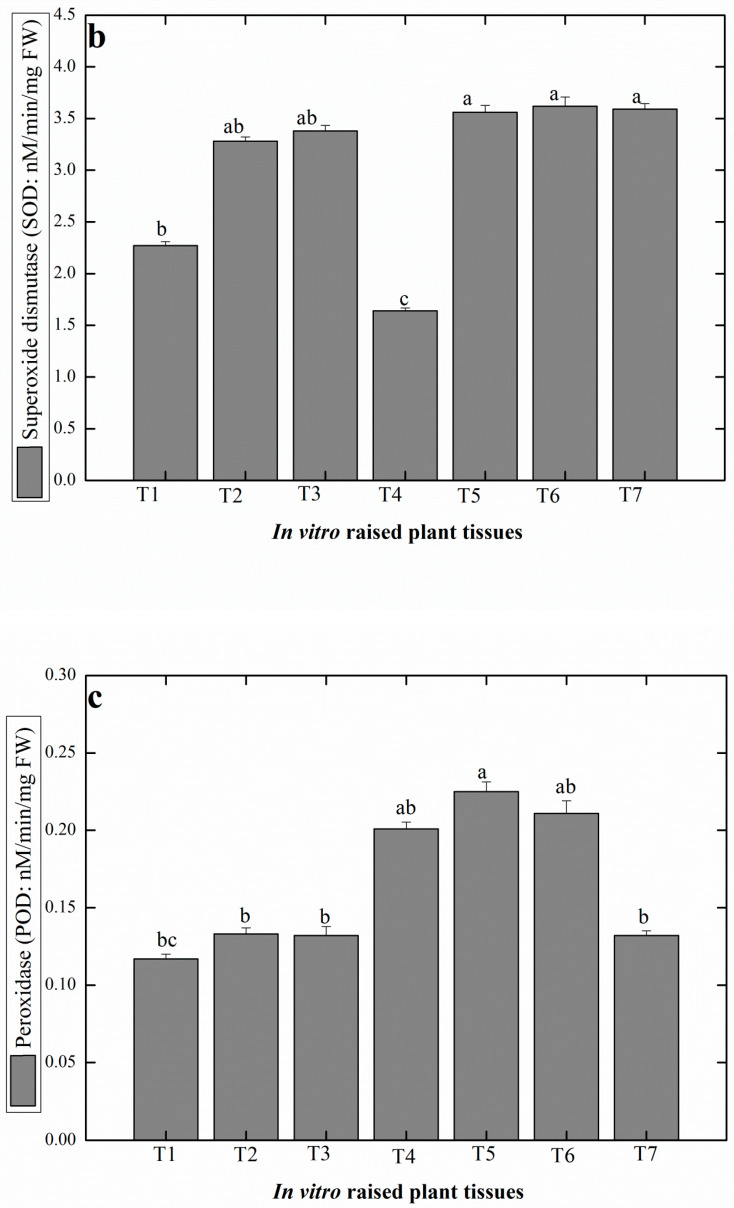

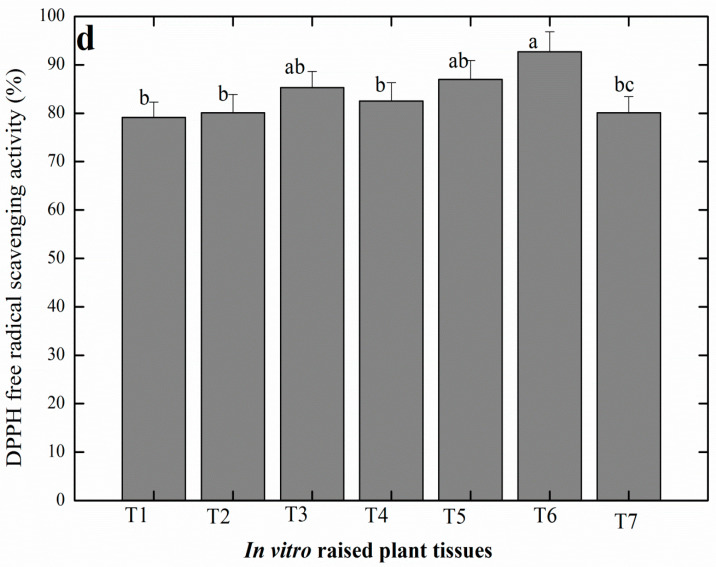
Estimation of the accumulation of antioxidant secondary metabolites in the micropropagated shoots of *L. usitatissimum* in response to different PGR treatments. (**a**): total phenolic content (TPC), total flavonoid content (TFC) and phenyl alanine ammonia lyase (PAL) activity, (**b**): superoxide dismutase (SOD) activity, (**c**): peroxidase (POD) activity, and (**d**): DPPH free radical scavenging activity. T1: 0.5 mg/L TDZ, T2: 1.0 mg/L TDZ, T3: 1.5 mg/L TDZ, T4: 0.5 mg/L Kn, T5: TDZ+ Kn (0.5 mg/L, each), T6: 1.0 mg/L TDZ+ 0.5 mg/L Kn, and T7: 1.5 mg/L TDZ+ 0.5 mg/L Kn. Values represent mean data of each treatment with ± standard error. Different letter(s) on each column data is statistically significant at *p* ≤ 0.5.

In our analysis, maximum amount of TPC (34.33 ± 0.20 mg of GAE/g DW) and TFC (8.99 ± 0.023 mg of QE/g DW) were determined in the shoots grown in MS media supplemented with TDZ (1.0 mg/L) + Kn (0.5 mg/L), whereas the least level of TPC (23.59 ± 0.012 mg of GAE/g DW) and TFC (2.56 ± 0.024 mg of QE/g DW) were detected in Kn (0.5 mg/L) treated cultures (Figure 2a). Further, the results showed that shoots regenerated in cultures supplemented with TDZ in combination with Kn accumulated higher levels of TPC and TFC as compared to TDZ tested alone. In a similar study, optimum TPC (5.10 mg of GAE/g DW) and TFC (2.72 mg of QE/g DW) were produced in callus culture raised in presence of 2.0 mg/L TDZ in *L. usitatissimum* [51].

PAL is a prominent enzyme that helps in the activation of phenylpropanoid metabolism in plants [52]. In this study, optimum PAL activity (8.99 ± 0.023 mg U/g FW) was observed in response to TDZ (1.0 mg/L) + Kn (0.5 mg/L), whereas the shoots developed in MS media containing individual TDZ (0.5 mg/L) resulted in lowest PAL activity (3.63 ± 0.066 mg U/g FW) (Figure 2a). In our study, the optimum amount of SOD (3.62 ± 0.015 nM/min/mg FW) and POD (0.225 ± 0.021 nM/min/mg FW) were recorded in shoot cultures grown in presence of (TDZ (1.0 mg/L + Kn 0.5 mg/L) and (TDZ (0.5 mg/L + Kn 0.5mg/L), respectively (Figure 2b,c). These antioxidant enzymes are produced by plant cell under severe stress conditions to decrease the oxidative risks, catalyze the superoxide (O_2_^−^) by dismutation reaction and convert the superoxide (O_2_^−^) into molecular oxygen (O_2_) or hydrogen peroxide (H_2_O_2_) through oxygen metabolism [53,54]. Studies have shown that among all PGRs, TDZ at 1.2 ppm was highly effective for production of SOD (4.2 U/mg protein) and POD (2.8 U/mg protein), respectively, in the shoot culture of *A. bracteosa* [32].

Another study concluded that the callus induced from the stem explant of linseed in response of TDZ (0.5–5.0 mg/L), showed a negative correlation in the antioxidant enzymes activity [55]. In eastern white pine the POD level was not efficiently produced at the beginning of the culture but then increased at later stages during shoot bud development [56]. Nevertheless, the antioxidant potential in the regenerated linseed shoot cultures was analyzed by using DPPH free radical scavenging assay. As indicated in Figure 2d, the maximum antioxidant activity (92.7 ± 1.32%) was recorded in the in vitro shoots regenerated in MS media fortified with (TDZ 1.0 mg/L + Kn 0.5 mg/L). While lowest activity was observed in TDZ alone (0.5 mg/L) treated cultures. Similarly Anjum et al. [57], observed highest antioxidant activity (91.51%) in the callus cultures developed from the leaf explant of linseed grown in TDZ (2.0 mg/L) supplemented media.

### 2.5. Production of Medicinally Potent Lignans in Linseed

**Figure 3:**
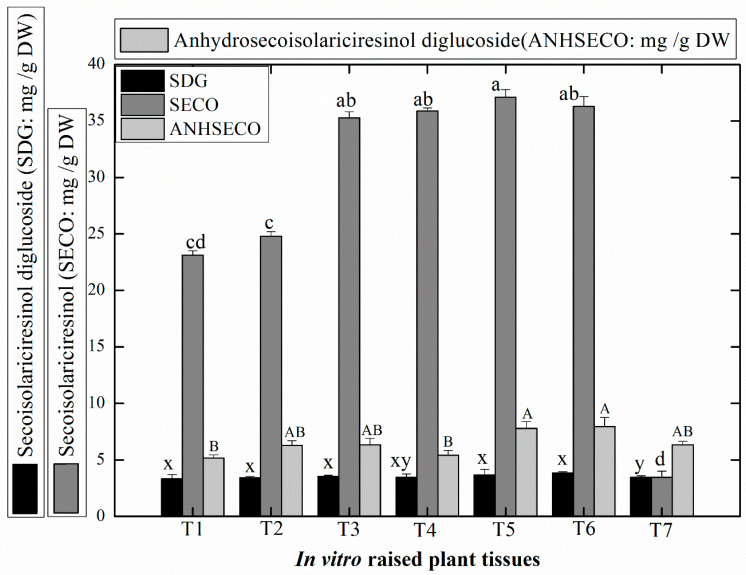
Estimation of the accumulation of bioactive lignans in the micropropagated shoots of *L. usitatissimum* in response to different PGR treatments. T1: 0.5 mg/L TDZ, T2: 1.0 mg/L TDZ, T3: 1.5 mg/L TDZ, T4: 0.5 mg/L Kn, T5: TDZ+ Kn (0.5 mg/L, each), T6: 1.0 mg/L TDZ+ 0.5 mg/L Kn, and T7: 1.5 mg/L TDZ+ 0.5 mg/L Kn. Values represent mean data of each treatment with ± standard error. Different letter(s) on each column data is statistically significant at *p* ≤ 0.5. SDG: (secoisolariciresinol diglucoside), SECO: (secoisolariciresinol), and ANHSECO (anhydrosecoisolariciresinol diglucoside).

In this study, considerable levels of pharmacologically active lignans such as secoisolariciresinol (SECO: 23.13–37.10 mg/g DW), secoisolariciresinol diglucoside (SDG: 3.32–3.86 mg/g DW), and anhydrosecoisolariciresinol diglucoside (ANHSECO: 5.15–7.94 mg/g DW) were accumulated in the shoot cultures (Figure 3). Higher amounts of SECO (37.10 ± 0.023 mg/g DW) were recorded in shoots grown over (TDZ + Kn (0.5 mg/L, each) treated MS media, whereas the lowest content of SECO (3.47 ± 0.013 mg/g DW) was detected in response to TDZ (1.5 mg/L)+ Kn (0.5 mg/L) treatment (Figure 3).

In general, *L. usitatissimum* shoots developed in response to TDZ and Kn synthesized optimum SDG and ANHSECO. Likewise, lower doses of these PGRs in combination were found suitable for optimum biosynthesis of SECO in the in vitro regenerated shoots of linseed. Several studies have reported that TDZ, in combination with other PGRs such as BA or NAA, has acted more effectively for maximum production of lignans in callus cultures of linseed [57,58,59].

Our results are in close agreement with findings of Aujum et al. [57], wherein TDZ (1.0 mg/L) in combination with NAA (2.0 mg/L) produced considerable levels of important lignans, i.e., SDG (0.7 ± 0.0045 mg/g DW) in leaf-derived callus cultures of linseed. In another study higher quantity of SECO was synthesized in response to fungal elicitation of linseed while no SDG was detected either in control or in elicited cell cultures [11]. In our study, the biosynthesis of lignans was dependent on the concentration of PGRs employed during micropropagation. Higher level of TDZ produced lower amounts of these metabolites. It is because of the synthesis of excessive ethylene production at elevated level of TDZ which suppresses the production of secondary metabolites [60].

Biosynthesis of lignans involves the metabolism of pinoresinol (furofuran), which is enantiomerically activated by dimerization of two coniferyl alcohol units with the aid of dirigent protein that results in the first product SDG (a cancer chemopreventive agent) in *Forsythia intermedia* [61]. Then a simultaneous reduction of this intermediate product via reductase enzyme (secoisolariciresinol dehydrogenase) forms dibenzoylbutane (secoisolariciresinol), followed by glycosylation of SECO via glycosyl transferase (a multigenic enzyme), and eventually results in the formation of SDG (secoisolariciresinol diglucoside) [62]. Many genes have been identified in linseed that encode SECO or SDG. In a study it was showed that maximum SDG was accumulated in the seed coat where optimum expression of UGT74S1 (uridine diphosphate glycosyltransferases (UGTs)) gene was detected in the seed coat, suggesting that it acts as SECO-GTs (Secoisolariciresinol glycosyltransferases) [62,63]. Some other studies showed that 2 GT (glycosyl transferase) genes, UGT74S1 and UGT94H, were highly expressed during the growth of the linseed and induced the expression of PLR (pinoresinol-lariciresinol reductase) genes, which play a significant role in SECO production. Thus, the UGT74S1 gene could synthesize mainly both lignans such as SECO and SDG [64]. Studies have reported that the two genes of linseed genome, encode the relative protein that activates the precursor (pinoresinol) for the formation of lignans during growth and development [61,65].

It is worth mentioning that SDG can be used as an anti-cancerous agent for the treatment of colon or breast cancer. It reduces the cancerous growth and cell division of the tumor cells through activating the apoptosis of the cancerous cells. SDG is also reported for its prominent anti-bacterial response against a diverse range of pathogens [66,67]. SDG and SECO have antioxidant potential, and are used to prevent the DNA from oxidative damage and lipid peroxidation. These lignans reduce the chances of metabolic syndrome by preventing the oxidative stress concerned with metabolism [8,68].

Further lignans such as SDG, ANHSECO, and SECO are also reported to lower the level of blood cholesterol (LDL and HDL) during glucose metabolism; thus, resulting in the prevention of cardiovascular diseases [69].

## 3. Materials and Methods

### 3.1. Plant Material, Sterilization and In Vitro Germination

The viable seeds of *L. usitatissimum* were first washed with sterile distilled water inside the laminar airflow hood, followed by immersion of seeds in 0.5% mercuric chloride (HgCl_2_) for 5 min, then washed with 70% ethanol (v/v) for 3 min. Finally, rinsed the seeds thoroughly 3 times with sterile distilled water. The seeds were then dried on a sterile filter paper and inoculated on autoclaved Murashige and Skoog medium (MS0) [70] media in test tubes containing sucrose 7.5 g/L and agar 0.8%. The PH of the media was adjusted to 5.8 before autoclaving. Finally, the test tubes were placed in the growth room for 16 h light and 8 h dark photoperiod condition and temperature at 25 ± 2 °C.

### 3.2. Optimization of In Vitro Conditions for Micropropagation and Plantlet Development

Initially, different explants (roots, hypocotyl, leaves, and cotyledon) were excised (1 cm in size) from 3 week old germinated shootlets. Based on the optimal response during shoot induction, hypocotyl explants were selected for subsequent experiments. Hypocotyl explants were cultured in the MS media containing TDZ either alone at concentration of 0.5–1.5 mg/L or Kinetin (Kn) at 0.5 mg/L and in combination with TDZ (0.5–1.5 mg/L) in the culture tubes containing sucrose 7.5 g/L and agar 0.8%. The pH of the media was adjusted to 5.8 before autoclaving. Finally, the test tubes were placed in the growth room for 16 h light and 8 h dark photoperiod condition and temperature at 25 ± 2 °C. The same level of each PGRs tested for shoot induction were also selected for shoot multiplication and elongation.

For each treatment, 50 test tubes were inoculated with the respective explants and the experiments were repeated. Observations were visually made in the cultures after a month culture period. However, shoot multiplication and elongation were recorded as the number of shoot per explant (mean) and shoot length (cm). The elongated shoots were derived from the shooting media and were further sub-cultured for root initiation and proliferation in the rooting media. The rooting media consisted of half or full-strength MS media, supplemented with various concentrations of indole butyric acid (IBA) at (0.1 mg/L, 0.5 mg/L, and 1.0 mg/L).

For the acclimatization process, plastic cups, with a small hole at the bottom were filled with a mixture of the 1–3 ratio of sand and field soil. To provide moist soil for better plant growth, all the cups were kept in the bowl containing water for 24 h to absorb water up to some limit. The rooted plants were removed from the test tubes and were carefully washed with sterile distilled water to remove the media. The plantlets were then transferred to small plastic cups and each cup was covered with thin plastic to avoid dryness. Finally, all the plants were placed in controlled conditions at growth room for 3 weeks and then transferred to the greenhouse.

### 3.3. Phytochemical Analysis

For assessment of the production of secondary metabolites in the shoot cultures, plant samples were harvested from the in vitro cultures established in the presence of different PGR treatments. Plant tissues from each growth treatment was harvested when the cultures were at their fully developed stages. Shoot cultures collected for phytochemical analysis were four week old. The plant samples were dried and crushed for phytochemical analysis. Extracts from the selected plant samples were subjected to different assays for the determination of the total phenolic content (TPC), total flavonoid content (TFC), and PAL based antioxidant activity.

#### 3.3.1. Total Phenolic Content

At the first step each selected dried sample was crushed and powder by the grinder. The crushed powder was mixed with 10 mL of 50% methanol and the solution was placed on a shaker at 24 rpm for 24 h at 25–30 °C, then sonication of the mixture was conducted for 30 min followed by vortexing for 30 min. Finally, centrifuging the solution at 6500 rpm for 15 min and removing the supernatant. The TPC was analyzed by using the protocol of Ali et al. [32], wherein a 20 μL of each extracted sample was added to 90 μL of 10× diluted Folin–Ciocalteu reagent. The mixture was then poured into the wells of a 96 well plate and incubated for 5 min. To maintain the final volume of the mixture at 200 μL, a further 90 μL of sodium carbonate was added to it.

For control treatment, same procedure was repeated in which methanol (20 μL) and gallic acid (1 mg/mL) were used as negative and positive control, respectively. The mixture was then centrifuged at 10,000 rpm for 13 min and filtered through a 45 μm thick membrane in UV visible spectrophotometer (Shimadzu-1650; Japan) cuvette. The absorbance was recorded at 630 nm after 90 min. For plotting, standard calibration curve gallic acid (Sigma; 1.0–10 mg/mL; R^2^ = 0.9878) was used. The results were expressed as mg gallic acid equivalent (GAE) per gram dry weight of extract.

#### 3.3.2. Total Flavonoid Content

For TFC determination, the method developed by Chang et al. [71] was used. For this purpose, 20 μL sample from 10 mg/mL of each sample was poured into wells of micro-plates, then 10 μL of aluminum chloride (10 g/L; d-H_2_O) and 10 μL of potassium acetate (98.15 g/L; d-H_2_O) was added to the reaction solution in microplate wells. For the control treatment, methanol (20 μL) and quercetin (1 mg/mL) were used as negative and positive control respectively. To maintain the final volume at 200 μL of the mixture, 160 μL d-H_2_0 was added. After 30 min, absorbance was recorded at 450 nm. The results were expressed as mg quercetin equivalent (QE) per gram dry weight of extract.

#### 3.3.3. Analysis of Antioxidant Enzyme Activities

For antioxidant enzymatic activity estimation, we used the protocol of Khan et al. [49] with some modification. Briefly, 0.2 g fresh plant extract of each sample was crushed by the grinder and 2 mL of phosphate buffer (50 mM, pH 7.8, and 0.1 mM EDTA) was added to the extract for homogenate formation. Then centrifugation of the mixture at 4 °C for 15 min at 12,000 rpm was followed. The supernatant was then removed and antioxidant enzyme activities were performed by using UV–vis spectrophotometer. Superoxide dismutase activity was determined by Khan et al. [49]. An assay system consists of riboflavin, methionine, and NBT was used for the analysis of SOD in micro propagated tissues. The procedure was modified for good analysis of enzyme activities in the selected samples. A mixture of 1.3 μM riboflavin, 13 mM methionine, and 63μM NBT was prepared, then added 20 μL of plant extract to the mixture, then a 0.05 M of sodium carbonate (pH10.2), and a little amount of water was added to the mixture. The reaction took place in a chamber illuminated with a 30 W fluorescent lamp at 25 °C. The reaction was started by turning the fluorescent lamp on and switch off the lamp after 5 min. A blue color formazan produced by NBT photoreduction was measured as an increase in absorbance activity at 560 nm. The enzyme activity of SOD was denoted as g^−1^ fresh weight (FW) min^−1^.

#### 3.3.4. Phenylalanine Ammonia-Lyase (PAL) Activity

PAL content in the cultured shoot was determined by the protocol of Ali et al. [72]. About 80–100 mg of each powder shoot sample was mixed with 100 mM potassium borate (ice cold buffer of pH 8.8) then added 2 nM of mercaptoethanol to the mixture. Following centrifugation the mixture at 12,000 rpm for 10 min at 4 °C, the supernatant was removed from the sample and was used for PAL assay, wherein 0.5 mL of supernatant from each sample was added to the tube already filled with 0.5 mL of phenylalanine (con: 4.0 mM^−1^) and 1.0 mL of potassium borate (BK_3_O_3_) buffer. The volume of the reaction mixture was maintained at 2.0 mL. The mixture was supplemented with 0.2 mL of 6 M HCl and then incubated at 30 °C for 30 min. Finally, the absorbance of the reaction mixture was recorded at 290 nm. Absorbance of the mixture occurred after the formation of the enzyme product which was proposed as one unit of the phenylalanine ammonia-lyase activity (U) as the amount of absorbance variation of 0.01.

#### 3.3.5. Peroxidase (POD) Activity

For measuring peroxidase activity of the selected samples, a 3.0 mL mixture was prepared by adding a 10 µL volume of enzyme solution to 2.99 mL of sodium phosphate buffer (50 mM, pH 6.0) containing 18.2 mM guaiacol and 4.4 mM hydrogen peroxide (H_2_O_2_) as substrates. POD activity was measured by calculating the deviation in absorbance at 470 nm at 25 °C. POD activity was defined as the amount of enzyme that caused an increase in absorbance at 470 nm of 0.001 per minute [73].

#### 3.3.6. Determination of Free Radicle Scavenging Activity

DPPH assay (2, 2-Diphenyl-1-picrylhydrazyl) was performed by using the method developed by Abbasi et al. [74]. Briefly, a mixture of 180 μL of DPPH (3.2 mg/100 mL methanol) and 20 μL of the extracted sample was prepared and then incubated for 30 min at room temperature (25 ± 2 °C). A methanolic extract of each sample in the DPPH solution (0.5 mL) was analyzed through UV–visible spectrophotometer. Absorbance of the reaction mixture was measured at 517 nm. Lastly, the radical scavenging activity was calculated as % of DPPH discoloration using the following equation.

% Free radical scavenging activity (% FRSA) = 100 × (1 − AC/AS)

where AC is absorbance of the solution when a sample extract was added at a particular concentration and AS is the absorbance of the DPPH solution (standard).

### 3.4. Analysis of Lignans in the Micro Propagated Shoots of L. Usitatissimum

The selected extracts from in vitro raised *L. usitatissimum* shoots were mixed with 1 mL (4:1 v/v) of the mixture of pyridine and BSTFA and added with 1% trimethylchlorosilane at 70 °C for 60 min to synthesize trimethylsilyl derivatives of lignans. For precise calibration, standards of SECO and ANHSECO were derivatized in the same pattern according to the method of Sarajlija et al. [75]. The analyzer system consisted of Shimadzu 2010 gas chromatography connected with an auto sampler (AOC-20i) and QP-2010 plus mass selective detector for GC/MS analysis. A 30 m HP-5 ms capillary column of 0.25 mm i.d. and 0.25 µm film thickness was used. After drying out of the mixture of pyridine-BTSFA-trimethylchlorosilane the salinized extracts were solubilized with hexane. Then 1 µL of the extracted sample was injected via injection port operated in uniform mode at 285 °C, for 30 s. The initial temperature of the column was kept at 175 °C. After 6 min it was raised up to 270 °C at 15 °C/min rate and held for 15.6 min (total run time was 28 min). The interface temperature was 285 °C. The total ion current (TIC) modal and/or selected ion monitoring (SIM) mode was used for analysis. For accurate and authentic determination of lignans, comparative analysis of the extracted lignans with the known measured reference standards was carried out by means of the combination of their chromatographic retention times and the ratio of mass ions [75].

### 3.5. Statistical Analysis

All the experiments were carried out by using a completely randomized design (CRD) with three replicates. The final data were statistically analyzed by one-way analysis of variance by using SPSS software. The significant difference in the comparison of means was shown with Duncan’s multiple range test (DMRT).

## 4. Conclusions

This is the first report on simple, rapid, and feasible micropropagation of *L. usitatissimum* and biosynthesis of medicinally potent lignans Optimum shoot regeneration was induced in the hypocotyl explants as compared with other explants. The combination of TDZ and Kn was more effective for direct shoot regeneration. Higher quantities of phenolics, flavonoids, antioxidant enzymes, and lignans were found in the shoot cultures treated with higher levels of TDZ. This study has a potential for promising production of biological active antioxidant metabolites and lignans.

## Figures and Tables

**Table 1:** Selection of suitable explant type and plant growth regulators (PGRs) for micropropagation through shoot organogenesis in *L. usitatissimum*. Values represent mean data of each treatment with ± standard error. Data was collected after four weeks culture period. Data with different letter(s) is statistically significant at *p* ≤ 0.5. TDZ: thidiazuron, Kn: Kinetin.

Shoot Regeneration Frequency (%)					
PGRs	Concentration (mg/L)	Leaf Explants	Cotyledon Explants	Hypocotyl Explants	Root Explants
TDZ	0.5	20.54 ± 0.82 ^d^	15.45 ± 0.76 ^d^	86.54 ± 6.93 ^a^	12.54 ± 0.86 ^cd^
TDZ	1.0	44.87 ± 2.45 ^bc^	30.54 ± 1.17 ^c^	81.23 ± 6.91 ^ab^	19.59 ± 0.78 ^c^
TDZ	1.5	27.68 ± 0.93 ^c^	41.75 ± 2.12 ^bc^	56 ± 2.66 ^c^	28.59 ± 0.95 ^bc^
Kn	0.5	57.76 ± 3.74 ^a^	24.36 ± 0.90 ^cd^	64.65 ± 4.47 ^bc^	32.43 ± 1.33 ^b^
Kn	1.0	46.12 ± 2.62 ^b^	26.75 ± 0.92 ^cd^	67.23 ± 4.71 ^b^	28.65 ± 0.95 ^bc^
Kn	1.5	40.43 ± 2.05 ^bc^	32.80 ± 1.33 ^c^	57.87 ± 3.75 ^c^	16.86 ± 0.77 ^c^
TDZ + Kn	0.5:0.5	50.42 ± 3.01 ^ab^	42.23 ± 2.45 ^bc^	86.87 ± 6.93 ^a^	45.76 ± 2.54 ^a^
TDZ + Kn	1.0:1.0	59.43 ± 3.95 ^a^	56.54 ± 3.62 ^a^	82.54 ± 6.92 ^ab^	38.94 ± 1.84 ^ab^
TDZ + Kn	1.5:1.5	48.76 ± 2.85 ^b^	47.65 ± 2.75 ^b^	80.83 ± 6.01 ^ab^	44.70 ± 2.44 ^a^

**Table 2:** Optimization of shoot organogenesis in *L. usitatissimum*. Values represent mean data of each treatment with ± standard error. Data was collected after four weeks culture period. Data with different letter(s) is statistically significant at *p* ≤ 0.5. TDZ: thidiazuron, Kn: Kinetin, DW: Dry weight, and FW: Fresh weight.

PGRs	Concentration (mg/L)	Days to Shoot Initiation	Frequency of Shoot Initiation	Avg. No of Shoots	Max. Shoot Length	Avg. Shoot Length	Avg. FW of Callus Developed at the Base (mg)	FW (Full Plant) mg	DW (Full Plant) mg
TDZ	0.5	7.1 ± 1.05 ^d^	86.54 ± 6.93 ^a^	1.33 ± 0.38 ^cd^	2.9 ± 0.15 ^e^	2.76 ± 0.12 ^cd^	140 ± 10.32 ^d^	770 ± 7.34 ^de^	638.8 ± 5.3 ^de^
TDZ	1.0	5.2 ± 0.85 ^f^	81.23 ± 6.91 ^ab^	1 ± 0.11 ^d^	3.3 ± 0.28 ^de^	2.96 ± 0.14 ^c^	750 ± 7.63 ^b^	1591 ± 9.25 ^a^	1336 ± 2.3 ^a^
TDZ	1.5	7.8 ± 0.90 ^c^	56 ± 2.66 ^e^	1 ± 0.11 ^d^	3.5 ± 0.39 ^d^	3.25 ± 0.23 ^bc^	1033 ± 11.01 ^a^	1333 ± 9.13 ^b^	1161.7 ± 4.9 ^b^
Kn	0.5	10 ± 0.81 ^a^	64.65 ± 4.47 ^d^	1.66 ± 0.57 ^c^	4.2 ± 0.34 ^cd^	3.9 ± 0.26 ^b^	0 ± 0	189 ± 1.6 ^f^	128 ± 1.9 ^f^
TDZ + Kn	0.5:0.5	8.6 ± 1.21 ^b^	86.87 ± 6.93 ^a^	6.3 ± 0.36 ^a^	6.5 ± 0.54 ^a^	4.56 ± 0.25 ^a^	40 ± 5.77 ^f^	858 ± 5.7 ^c^	742.7 ± 2.5 ^c^
TDZ + Kn	1.0:0.5	6.5 ± 0.55 ^e^	82.54 ± 6.92 ^ab^	2 ± 0.25 ^bc^	4.6 ± 0.44 ^c^	4.15 ± 0.32 ^ab^	91.4 ± 7.63 ^e^	422.8 ± 0.013 ^e^	276 ± 6.4 ^e^
TDZ + Kn	1.5:0.5	7.3 ± 0.95 ^cd^	80.83 ± 6.01 ^ab^	2.5 ± 0.46 ^b^	5.5 ± 0.44 ^b^	4.25 ± 0.42 ^ab^	500 ± 12.58 ^c^	789.3 ± 4.6 ^d^	657.8 ± 2.4 ^d^

**Table 3:** Development of roots from the regenerated shoots in MS full or half strength medium with various concentrations of indole butyric acid (IBA) in *L. usitatissimum*. Values represent mean data of each treatment with ± standard error. Data was collected after four weeks culture period. Data with different letter(s) is statistically significant at *p* ≤ 0.5. MS0: MS media with no plant growth regulators.

Media	PGRs	Concentration (mg/L)	Frequency of Root Induction (%)	Days to Root Initiation	Avg. Root Length
Full strength MS media	IBA	0.1	27.6 ± 0.95 ^cd^	10 ± 0.81 ^b^	3.1 ± 0.21 ^b^
	IBA	0.5	36.9 ± 1.84 ^b^	10 ± 0.81 ^b^	3.1 ± 0.21 ^b^
	IBA	1.0	21.5 ± 0.82 ^d^	10 ± 0.81 ^b^	3.1 ± 0.21 ^b^
Half strength MS media	IBA	0.1	45.7 ± 2.54 ^a^	8 ± 0.20 ^c^	3.5 ± 0.46 ^a^
	IBA	0.5	42.2 ± 2.45 ^ab^	8 ± 0.20 ^c^	3.5 ± 0.46 ^a^
	IBA	1.0	32.43 ± 1.33 ^bc^	8 ± 0.20 ^c^	3.5 ± 0.46 ^a^
MS0	No PGRs	0	33.16 ± 1.34 ^c^	13 ± 0.95 ^a^	1.3 ± 0.16 ^c^
